# Ineffectiveness of immune checkpoint inhibitors in prostate cancer: is it just a question of ‘‐mabs’?

**DOI:** 10.1002/path.70095

**Published:** 2026-07-08

**Authors:** Giuseppe Schepisi, Irene Torresan, Emilio Francesco Giunta, Sara Bleve, Nicole Brighi, Elisa Tassinari, Sofia Zanuccoli, Maria Concetta Cursano, Cristian Lolli

**Affiliations:** ^1^ Department of Oncology Istituto Romagnolo per lo Studio dei Tumori ‘Dino Amadori’ (IRST) IRCCS ‐ Meldola (FC) Meldola Italy

**Keywords:** immunotherapy, immune checkpoint inhibitors, prostate cancer, immunosuppressive factors

## Abstract

Prostate cancer (PCa) is the second most common type of neoplasm, currently treated with a variety of therapeutic agents. Although advances in treatment over the past decade have improved overall survival, patients with advanced PCa eventually progress from hormone‐sensitive PCa to metastatic castration‐resistant PCa. For this reason, other therapeutic strategies, including immunotherapy, are being investigated. Despite initial enthusiasm, these approaches have not yet produced significant improvements in either progression free survival or overall survival. Several factors may have contributed to the consistently disappointing results of immune checkpoint inhibitors in PCa, despite their more successful results in other tumor types. In this review, we discuss the potential reasons for these disappointing clinical results. © 2026 The Author(s). *The Journal of Pathology* published by John Wiley & Sons Ltd on behalf of The Pathological Society of Great Britain and Ireland.

## Introduction

Even today, prostate cancer (PCa) remains the second most common type of neoplasm and the fifth major cause of cancer‐related death in males around the world [1]. Despite therapeutic advances over the past decade having prolonged overall survival (OS), patients with advanced PCa eventually progress from hormone‐sensitive PCa (HSPC) to metastatic castration‐resistant PCa (mCRPC). PCa is currently treated with various types of therapeutic agents, including hormone therapy, chemotherapy, radiometabolic therapies, and poly(ADP‐ribose) polymerase (PARP) inhibitors. Furthermore, as for other tumor histotypes, alternative therapeutic strategies are also being investigated, including immunotherapy. The first immunotherapeutic agent approved against PCa was Sipuleucel‐T. However, following the advent of new immune checkpoint inhibitors (ICIs), several molecules have been investigated as monotherapy, or in combination with chemotherapy, or as bispecific antibodies, or as chimeric antigen receptor (CAR)‐T‐cell therapy.

Despite initial enthusiasm, these attempts have not yet produced satisfactory results in terms of either progression free survival (PFS) or OS. Recently, in a compelling editorial published in the *Journal of Clinical Oncology*, Slovin [2], drawing on the disappointing results of the Keynote‐921 study [3] and similar previous trials, highlighted several factors that may have contributed to the consistently negative results of ICIs in PCa.

**Figure 1:**
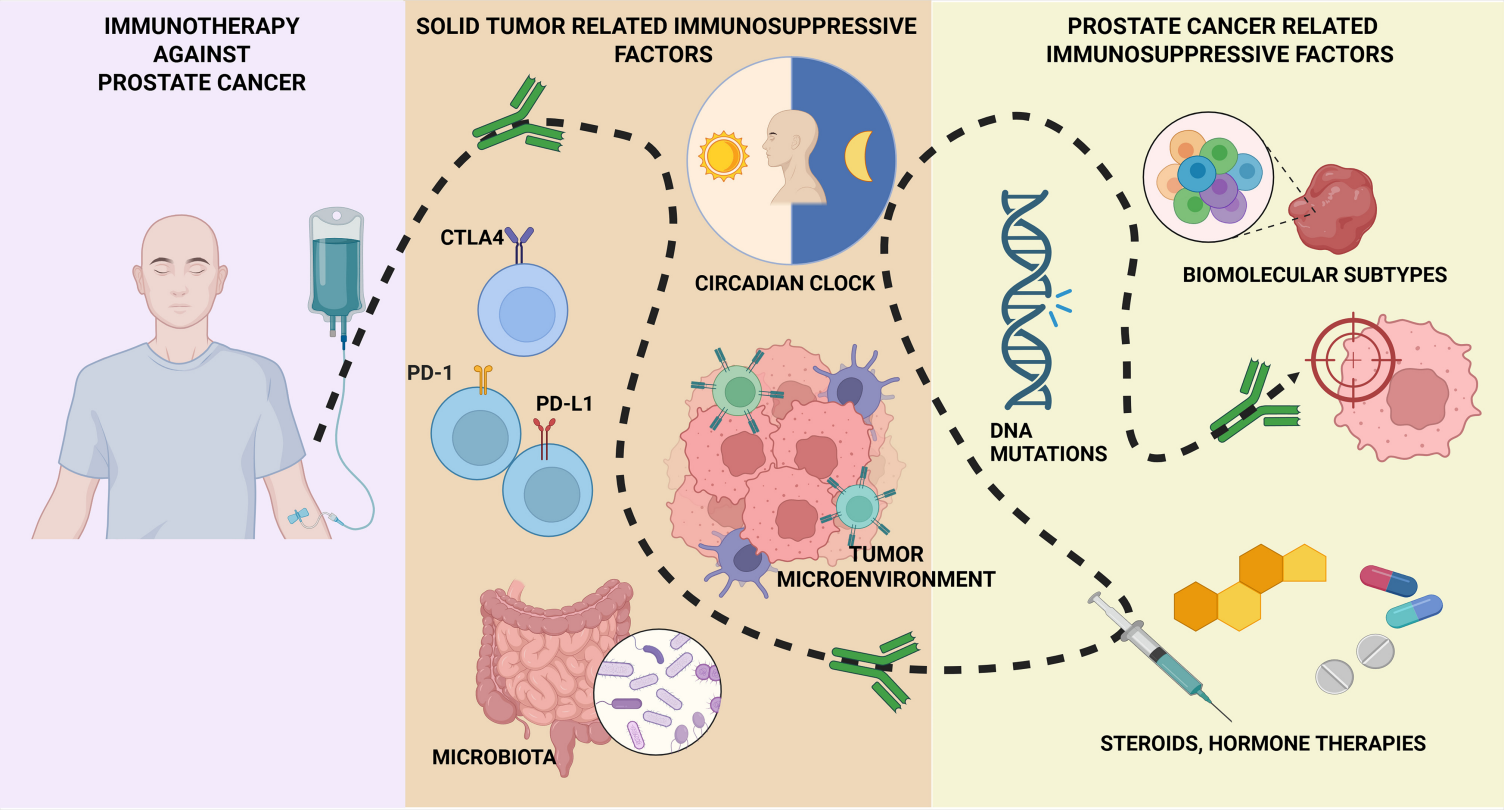
Potential factors involved in immune function modulation in prostate cancer (PCa). CTL4, cytotoxic T‐lymphocyte‐associated protein 4; PD‐1, programmed cell death 1; PD‐L1, programmed cell death ligand 1. Created in BioRender. Schepisi, G. (2025) https://BioRender.com/2pmfwhg.

We agree with these hypotheses; however we believe that other potential contributing factors should also be considered. In this review, we discuss several additional factors that may have contributed to these disappointing results (Figure 1).

## Preclinical rationale for immunotherapy in PCa

The use of immunotherapeutic agents in PCa presents a strong preclinical rationale, both as a monotherapy and in combination with conventional therapies.

Firstly, several studies have demonstrated that the tumor microenvironment (TME) in patients with PCa is immunosuppressive, with enrichment of cancer associated fibroblasts (CAFs), MMP‐2, cytokines and chemokines, regulatory T cells (Tregs), myeloid‐derived suppressor cells (MDSCs), and M2 macrophages, all of which may promote immune evasion [4]. For this reason, restoring the T‐cell antitumor response through immune checkpoint blockade may represent an intriguing treatment approach [5, 6]. In particular, programmed cell death ligand 1 (PD‐L1) hyperexpression on tumor‐infiltrating T cells has been associated with disease progression in patients with PCa [7].

Secondly, several studies have demonstrated that docetaxel can activate the cGAS‐STING pathway, leading to interferon signaling and induction of lymphocytic infiltrates in the tumor microenvironment (TME) [2]. This ability of docetaxel to remodulate cellular components within the TME could, in turn, lead to up‐regulation of programmed cell death 1 (PD‐1)/PD‐L1 expression, potentially increasing the sensitivity of PCa to immune checkpoint inhibitors [8].

Thirdly, other studies have shown that next‐generation hormonal agents also have immunomodulatory activity. For example, enzalutamide has been shown to stimulate activation of the interferon (IFN)‐ɣ pathway and remodel cellular components within the TME by reducing the presence of MDSCs [9]. Furthermore, in a recent immunohistochemical study, increased PD‐L1 expression and immunoreactivity was reported in metastatic sites from patients with PCa previously treated with androgen receptor pathway inhibitors (ARPIs) [10]. In this regard, other studies have confirmed that enzalutamide‐resistant cancer cells can up‐regulate the PD‐1/PD‐L1 pathway, thereby modulating T‐cell activity in TME [11, 12]. In light of these findings, it has been proposed that combining an ARPI with an immune checkpoint inhibitor may exert a synergistic effect and potentially resensitize PCa cells to immune‐mediated therapy.

More recently, several studies have demonstrated the efficacy of PARP inhibitors in PCa. PARP inhibition can lead to endocytoplasmic accumulation of DNA fragments due to unrepaired DNA damage, which in turn activates the DNA sensing cyclic GMP–AMP synthase (cGAS)–stimulator‐of‐interferon genes (STING) pathway, resulting in the production of type I interferons [13]. Thus, the antitumor activity driven by interferon production may be further enhanced by combination with an ICI, independent of BRCAness [14, 15].

## Main studies involving ICIs in PCa

Based on the above‐described rationale, numerous studies have investigated the use of ICIs in PCa (Table 1).

**Table 1:** Overview of key clinical trials evaluating the efficacy of ICIs in prostate cancer.

Trial	Phase	Number of patients (pts)	Setting	Intervention	Key efficacy endpoints	Biomarker evaluation
NCT01375842 (Petrylak DP, 2021) [16]	Ia	35	mCRPC with PD after enzalutamide or Sipuleucel‐T	Atezolizumab	Safety (primary endpoint): TRAEs ≥ G3 in 11.4% of pts PSA_50_ RR: 8.6% OS: 14.7 months	OS by presence* (*n* = 10) versus absence (*n* = 4) of biomarker: NR* ≥ 1 between: TMB ≥10 mut/Mb, DDR^+^, MSI‐H, PD‐L1 ≥ 5%
NCT03016312 (Powles T, 2022) [17]	III	759	mCRPC with PD after abiraterone and PD or ineligible for taxane regimen	Atezolizumab + enzalutamide (*n* = 397) versus enzalutamide (*n* = 380)	OS (primary endpoint): HR 1.12 (*p* = 0.28) rPFS: HR 0.9 Safety: TRAEs ≥ G3 in 28.3% (atezolizumab + enzalutamide) and in 9.6% (enzalutamide) of pts, 8 treatment‐related deaths	PFS in biomarker‐evaluable population (*n* = 750): PD‐L1 ≥ 5% (*n* = 38): HR 0.28 (*p* = 0.003) CD8^+^ T cells ≥ median (*n* = 281): HR 0.72 (*p* = 0.03) TMB ≥ median (2.25 mut/Mb) (*n* = 158): HR 0.67 (*p* = 0.03) DDR^+^ (*n* = 98): HR 0.79 (*p* = 0.33) PTEN loss (*n* = 75): HR 0.67 (*p* = 0.04)
NCT00861614 (CA184‐043) (Kwon ED, 2014) [18]	III	799	mCRPC PD after docetaxel	Bone‐directed RT (8 Gy in 1 fraction) followed by either ipilimumab 10 mg/kg or placebo every 3 weeks for up to 4 doses	OS: 11·2 months with ipilimumab versus 10·0 months with placebo (HR 0·85, 0·72–1·00; *p* = 0·053) HR changed over time: HR 0–5 months 1.46 HR 5–12 months 0.65 HR beyond 12 months 0.60	NA
NCT01057810 (CA184‐095) (Beer T, 2017) [19]	III	602	CT‐naive pts with mCRPC without visceral metastases	Ipilimumab 10 mg/kg or placebo every 3 weeks for up to 4 doses Ipilimumab 10 mg/kg or placebo maintenance to pts with non‐progression every 3 months	Median OS (primary endpoint) 28.7 months with ipilimumab versus 29.7 months with placebo (*p* = 0.3667)	PSA reduction: 23% ipilimumab 8% placebo
NCT02985957 (Sharma P, 2020) [20]	II	90	mCRPC Cohort 1 (*n* = 45): PD after ≥1 NHT, CT‐naïve in mCRPC Cohort 2 (*n* = 45): PD after CT in mCRPC	Nivolumab (1 mg/kg) + ipilimumab (3 mg/kg)	ORR in cohort 1 (primary endpoint): 25% ORR in cohort 2 (primary endpoint): 10% rPFS in cohort 1 (primary endpoint): 5.5 months rPFS in cohort 2 (primary endpoint): 3.8 mo OS in cohort 1: 19 mo OS in cohort 2: 15.2 mo Safety: TRAEs ≥ G3 in 42% (cohort 1) and 53% (cohort 2) of pts, 4 treatment‐related deaths	ORR by: TMB ≥ median (74.5 mut/pts) versus < median: 50% versus 5.3% HRD^+^ versus HRD^−^: 50% versus 22.6% DDR^+^ versus DDR^−^: 36.4% versus 23.1% PD‐L1 ≥ 1% versus PD‐L1 < 1%: 36.4% versus 12.1%
NCT04717154 (van Wilpe S, 2024) [21]	II	69	mCRPC molecularly selected (i.e. dMMR or nsTMB ≥7.1 mut/Mb or BRCA2 mutation or biallelic CDK12 inactivation) Cohort A (*n* = 65): ICI‐naïve Cohort B (*n* = 4): prior ICI monotherapy	Nivolumab (3 mg/kg) + ipilimumab (1 mg/kg)	6‐month DCR (primary endpoint): 38% ORR: 38% PSA_50_ RR: 47% Safety: TRAEs ≥ G3 in 48% of pts, 2 treatment‐related deaths	6‐month DCR by: dMMR (*n* = 21): 81% nsTMB ≥7.1 mut/Mb (*n* = 8): 25% *BRCAm* (*n* = 20): 15% *CDK12i* (*n* = 16): 19%
NCT03061539 (NEPTUNES) (Leone G, 2025) [22]	II	71	ImS+ mCRPC in PD after ≥1 treatment line ImS+ defined by (1) dMMR (2) DNA damage repair gene loss and/or (3) HII	Nivolumab (1 mg/kg) + ipilimumab (3 mg/kg) (C1) or nivolumab (3 mg/kg) + ipilimumab (1 mg/kg) (C2) followed by nivolumab 480 mg every 4 weeks up to 10 cycles	CRR (primary endpoint) 23/71 (32%) RRs higher in pts with dMMR (7/10), BRCA2 loss (4/8) HII ± other ImS+ features (13/30). Duration of response for HII without other ImS+ 2.6 months, DNA repair gene loss without dMMR 17.3 months dMMR 10 months	NA
NCT03338790 (Fizazi K, 2021) [23]	II Cohort B	84	mCRPC CT‐naïve, ≤ 2 NHTs allowed	Nivolumab + docetaxel	ORR (primary endpoint) (*n* = 45): 40% PSA_50_ RR (primary endpoint) (*n* = 81): 46.9% Safety (*n* = 84): TRAEs ≥ G3 in 47.6% of pts, 3 treatment‐related deaths mCRPC with prior NHTs (*n* = 31): ORR: 38.7% PSA_50_ RR: 39.6% mrPFS: 8.5 months mOS: 16.2 months	HRD^+^ (*n* = 19): ORR: 36.8% PSA_50_ RR: 50% TMB ≥10 mut/Mb (*n* = 13): ORR: 38.5% PSA_50_ RR: 31.6%
NCT02787005 (Antonarakis ES, 2020) [24]	II	258	mCRPC with PD after ≥ 1 NHT and ≤ 2 CT lines Cohort 1 (*n* = 133) and 2 (*n* = 66): RECIST‐measurable PD‐L1^+^ (CPS ≥1) and PD‐L1^−^ disease, respectively Cohort 3 (*n* = 59): bone predominant disease	Pembrolizumab	ORR in cohort 1 and 2 (primary endpoint): 5% ORR in cohort 1 (primary endpoint): 5% ORR in cohort 2 (primary endpoint): 3% OS in cohort 1: 9.5 months OS in cohort 2: 7.9 months OS in cohort 3: 14.1 months Safety: TRAEs ≥ G3 in 15% of pts, 2 treatment‐related deaths	ORR by: BRCA1/2 or ATM aberrations (*n* = 19): 11% HRD^+^ (*n* = 10): 0%
NCT02312557 (Graff JN, 2020) [25]	II	28	mCRPC with PD after enzalutamide	Pembrolizumab + enzalutamide	PSA_≥50_ RR (primary endpoint): 18% ORR (*n* = 12): 25% OS: 22.2 months Safety: G3 irTRAEs in 7% of pts	NA
NCT03834493 (Graff JN, 2023) [26]	III	1,244	mCRPC CT‐naive in mCRPC, PD after abiraterone or no prior abiraterone	Pembrolizumab + enzalutamide (*n* = 621) versus PBO + enzalutamide (*n* = 623)	OS (primary endpoint): HR 0.98 (*p* = 0.41) rPFS (primary endpoint): HR 0.66 (*p* = 0.66) Safety: TRAEs ≥ G3 in 31.2% (pembrolizumab + enzalutamide) and in 10.8% (PBO + enzalutamide) of pts, 3 treatment‐related deaths	NA
NCT04191096 (Gratzke CJ, 2023) [27]	III	1,251	mHSPC no prior NHTs, allowed prior docetaxel (≤ 6 cycles)	Pembrolizumab + enzalutamide (*n* = 626) versus PBO + enzalutamide (*n* = 625)	OS (primary endpoint): HR 1.16 rPFS (primary endpoint): HR 1.2 (*p* = 0.95) Safety: TRAEs ≥ G3 in 41.8% (pembrolizumab + enzalutamide) and in 13.9% (PBO + enzalutamide) of pts	NA
NCT02861573 (Yu EY, 2022) [28]	Ib/II Cohort B	105	mCRPC CT‐naïve, PD after abiraterone or enzalutamide	Pembrolizumab + docetaxel	Safety (primary endpoint): RAEs ≥ G3 in 44% of pts, 2 treatment‐related deaths PSA RR (primary endpoint) (*n* = 103): 34% ORR (primary endpoint) (*n* = 52): 23% rPFS: 8.5 mo OS: 20.2 mo	PSA RR by: PD‐L1 CPS ≥1 (*n* = 24): 67% PD‐L1 CPS < 1 (*n* = 75): 75%
NCT03834506 (Petrylak DP, 2025) [3]	III	1,030	mCRPC with PD after one previous NHT in mHSPC or mCRPC, allowed prior docetaxel (≤ 6 cycles) in mHSPC	Pembrolizumab + docetaxel (*n* = 515) versus PBO + docetaxel (*n* = 515)	OS (primary endpoint): HR 0.92 (*p* = 0.17) rPFS (primary endpoint): HR 0.85 (*p* = 0.03) Safety: TRAEs ≥ G3 in 43.2% (pembrolizumab + docetaxel) and in 36.6% (PBO + docetaxel) of pts, 9 treatment‐related deaths	OS by: PD‐L1^+^ (*n* = 126 versus 102): HR 0.71 PD‐L1^−^ (*n* = 346 versus 372): HR 0.99
NCT03834519 (Antonarakis ES, 2023) [29]	III	793	mCRPC with PD after abiraterone in mHSPC/mCRPC or enzalutamide in mCRPC and on docetaxel	Pembrolizumab + olaparib (*n* = 529) versus NHTs (i.e. abiraterone or enzalutamide, *n* = 264)	OS (primary endpoint): HR 1.02 (*p* = 0.55) rPFS (primary endpoint): HR 0.94 (*p* = 0.26) Safety: TRAEs ≥ G3 in 35% (pembrolizumab + olaparib) and in 9% (NHTs) of pts, 4 treatment‐related deaths	HRR mutated (*n* = 138 versus 59): rPFS: HR 0.69, OS HR 0.88 HRR non‐mutated (*n* = 355 versus 173): rPFS: HR 1.26, OS HR 0.97 PD‐L1 CPS ≥1 (*n* = 127 versus 50): rPFS: HR 1.03, OS HR 0.90 PD‐L1 CPS < 1(*n* = 365 versus 197): rPFS: HR 1.07, OS HR 0.94
NCT03204812 (Subudhi SK, 2021) [30]	II	26	CT‐naïve metastatic (bone) CRPC	Tremelimumab (75 mg) + durvalumab (1,500 mg) every 4 wks (up to 4 doses) prior to durvalumab (1,500 mg) maintenance every 4 wks (up to 9 doses)	TRAEs (primary endpoint) in 11 pts (42%), no grade 4 or 5 events, discontinued in 3 pts (12%) SD in 6 pts (24%) for >6 months mrPFS (43 mo) 3.7 mo mOS 28.1 mo	PSA decline ≥50% in 3 pts (12%)
NCT04446117 (Agarwal N, 2025) [31]	III	575	mCRPC CT‐naïve, ≤ 1 NHT	Atezolizumab + cabozantinib (*n* = 289) versus NHTs (i.e., abiraterone or enzalutamide, *n* = 286)	OS (primary endpoint): HR 0.89 (*p* = 0.30) PFS (primary endpoint): HR 0.65 (*p* < 0.001) Safety: TRAEs ≥ grade 3 in 40% (atezolizumab + cabozantinib) and in 8% (NHTs)	NA

*CDK12i*, *CDK12* inactivating mutations; CPS, combined positive score; CRR, composite response rate; CT, chemotherapy; DCR, disease control rate; DDR, DNA damage repair; dMMR, deficient DNA mismatch repair; G3, grade 3; HII, high inflammatory infiltrate; HRD, homologous recombination deficiency; HRR, homologous recombination repair; ICI, immune checkpoint inhibitor; ImS+, immunogenic signature–positive MSI‐H, microsatellite instability‐high; irTRAEs, immune‐related treatment‐related adverse events; mCRPC, metastatic castration‐resistant prostate cancer; mHSPC, metastatic hormone‐sensitive prostate cancer; NA, not applicable; NE, not evaluable; NHTs, novel hormonal therapies; NR, not reported; nsTMB, non‐synonymous tumor mutational burden; ORR, overall response rate; OS, overall survival; PBO, placebo; PD, progressive disease; PD‐L1, programmed cell death ligand 1; PFS, progression‐free survival; pts, patients; PTEN, phosphatase and tensin homolog; PSA_50_ RR, ≥ 50% decrease in PSA from baseline; rPFS, radiographic progression‐free survival; RT, radiotherapy; TMB, tumor mutational burden; TRAEs, treatment‐related adverse events; PSA RR, PSA response rate.

In 2010, the multicenter Immunotherapy for Prostate AdenoCarcinoma Treatment (IMPACT) phase III trial demonstrated that Sipuleucel‐T (an autologous active cellular immunotherapy) extended OS by 4.1 months in a cohort of 512 patients with mCRPC compared with the placebo group. However, no significant difference was observed in median time to objective disease progression between the two patient cohorts [32]. Based on these results, Sipuleucel‐T was approved by the FDA in 2010 for the treatment of asymptomatic or minimally symptomatic mCRPC.

The approval of Sipuleucel‐T, along with growing interest in immune checkpoint inhibitors, has led the scientific community to investigate new immunotherapies for PCa.

The pivotal CA184‐043 phase III study evaluated anti‐cytotoxic T‐lymphocyte‐associated protein 4 (CTLA‐4) ipilimumab versus placebo after bone‐directed radiotherapy in a cohort of 799 patients with mCRPC whose condition had progressed following docetaxel. Although the study did not demonstrate any differences between the two groups in terms of OS, the hazard ratio progressively improved over time in favor of ipilimumab, demonstrating some activity of this treatment, thus warranting further in‐depth studies [18]. A subsequent study (CA184‐095 trial) tested ipilimumab versus placebo in a cohort of 602 patients with metastatic castration‐resistant prostate cancer without visceral metastases. Again, ipilimumab demonstrated no improvement in OS, but showed an advantage over placebo in terms of PSA reduction (23% versus 8%, respectively), suggesting a benefit in a subset of patients [19]. For this reason, Gao *et al* conducted a translational study to investigate the mechanisms of ipilimumab resistance, demonstrating that the levels of PD‐L1 and VISTA were increased in distinct macrophage subsets following ipilimumab treatment. The authors focused on the role of VISTA as a compensatory pathway potentially responsible for the limited activity of anti‐CTLA‐4 inhibitor ipilimumab in patients with mCRPC [33].

A meta‐analysis of 13 studies involving 2,533 patients with mCRPC reported that anti‐PD‐1/PD‐L1 combination therapy significantly improved PFS, although OS in patients with CRPC requires further evaluation [34]. To date, no ICI‐based combination has shown superiority over standard treatment in a randomized clinical trial.

In a phase Ia study, 35 patients with mCRPC who were pretreated underwent atezolizumab monotherapy, with a PSA response rate of 8.6% [16].

The IMbassador250 trial (NCT03016312 https://clinicaltrials.gov/study/nct03016312) investigated a combination of PD‐L1 inhibitor atezolizumab plus enzalutamide versus enzalutamide alone in 759 patients with mCRPC whose condition had progressed on abiraterone. The combination treatment did not demonstrate an OS benefit in unselected patients. Subsequent molecular analyses conducted in this study revealed no correlation between PD‐L1 status and treatment response, nor between DDR alterations and PFS. In contrast, a correlation was observed between high tumor mutational burden (TMB) – rarely found in PCa – and oncological response, suggesting the need for careful selection of patients who can benefit from immunotherapy, whether as monotherapy or in combination [17].

A phase II CheckMate 650 trial (NCT02985957 https://clinicaltrials.gov/study/NCT02985957) examined the combination of anti‐PD‐1 nivolumab and anti‐CTLA‐4 ipilimumab. This combination achieved a benefit both in pre‐ and post‐chemotherapy cohorts in terms of objective response rate (ORR) (25% and 10% respectively) and median OS (19.0 and 15.2 months, respectively). However, the combination resulted in a high incidence of severe treatment‐related adverse events (TRAEs), including four treatment‐related deaths [20].

More recently, the same immune combination (i.e., ipilimumab 1 mg/kg plus nivolumab 3 mg/kg every 3 weeks for four cycles, followed by nivolumab 480 mg every 4 weeks for up to 52 weeks) was evaluated in a cohort of 69 patients with mCRPC, recruited according to their molecular status (INSPIRE trial) [21]. The trial met its primary endpoint, reporting a DCR > 6 months in 38% of cases. Only limited responses were observed in the *CDK12* inactivating mutations (*CDK12*i), BRCA‐mutated (BRCAm), and TMB‐high subgroups, whereas remarkable efficacy was reported in the mismatch repair deficiency (dMMR) subgroup. A high incidence of TRAEs was also reported in this study.

In a single‐arm pilot study, Subudhi *et al* tested a combination therapy of tremelimumab plus durvalumab every 4 weeks (up to four doses), followed by durvalumab maintenance every 4 weeks (up to nine doses) in a cohort of 26 chemotherapy‐naïve patients with mCRPC. The combination treatment was well tolerated and demonstrated potential activity in a subset of patients [30].

Results from the NEPTUNES study, which evaluated a combination of nivolumab and ipilimumab in patients with mCRPC and a positive immunogenic signature (ImS+), were recently published. ImS+ was defined by (1) dMMR; (2) DNA damage repair gene loss; and/or (3) high tumor‐infiltrating lymphocytes (TILs) (HII). Patients received four cycles of nivolumab 1 mg/kg plus ipilimumab 3 mg/kg or nivolumab 3 mg/kg plus ipilimumab 1 mg/kg, followed by nivolumab 480 mg monthly up to 10 cycles. The authors demonstrated that nivolumab 1 mg/kg plus ipilimumab 3 mg/kg was efficacious in ImS+ pretreated mCRPC, whereas the alternative schedule (nivolumab 3 mg/kg plus ipilimumab 1 mg/kg) was associated with less toxicity but potentially reduced activity [22].

In a cohort of the phase II CheckMate 9KD study, 54 patients pretreated only with one or two previous ARPI regimens but not with chemotherapy, received combination treatment with nivolumab plus docetaxel. In the 31 evaluable cases in this cohort, the authors reported an ORR of 38.7%, a median radiographic PFS (rPFS) of 8.5 months, and a median OS of 16.2 months [23].

In cohorts 1–3 of the KEYNOTE‐199 trial, pembrolizumab monotherapy demonstrated durable antitumor activity and an acceptable safety profile in chemotherapy‐ and hormone‐pretreated patients with mCRPC [24].

The intriguing results obtained led to testing pembrolizumab in combination with ARPIs. However, although a single‐arm phase II study conducted on a small cohort of 28 patients with mCRPC progressed on enzalutamide had shown some efficacy from the combination [25], two subsequent studies have denied these results. In fact, two randomized double‐blind phase III trials evaluated anti‐PD‐1 pembrolizumab combined with enzalutamide versus enzalutamide plus placebo in 1,251 patients with mHSPC (KEYNOTE‐991; NCT04191096 https://clinicaltrials.gov/study/NCT04191096) and 1,244 patients with mCRPC (KEYNOTE‐641; NCT03834493 https://clinicaltrials.gov/study/NCT03834493), respectively. Both studies were terminated for futility [26, 27].

In cohort B of the phase Ib/II KEYNOTE‐365 trial (NCT02861573 https://clinicaltrials.gov/study/NCT02861573), the authors evaluated the safety, tolerability, and efficacy of pembrolizumab in combination with docetaxel (75 mg/m^2^) and prednisone (5 mg twice daily) in chemotherapy‐naïve, hormone‐pretreated patients with mCRPC. A PSA response rate of 34%, an ORR of 23%, a median rPFS of 8.5 months, and median OS of 20.2 months were reported. Grade 3–5 TRAEs occurred in 44% of cases, with seven AE‐related deaths (two due to treatment‐related pneumonitis) [28].

Recently, in the phase III KEYNOTE‐921 study (NCT03834506 https://clinicaltrials.gov/study/NCT03834506), pembrolizumab combined with docetaxel and prednisone or prednisolone did not improve OS in patients with CRPC [3].

The randomized open‐label phase III KEYLYNK‐010 study evaluated the combination of pembrolizumab with PARP inhibitor olaparib versus ARPI in a cohort of 793 pretreated patients with mCRPC. However, the study was terminated for futility [29].

Recently, the CONTACT‐02 study tested the combination of tyrosine kinase inhibitor cabozantinib with anti‐PD‐L1 atezolizumab compared with ARPI in a cohort of chemo‐naive patients with mCRPC (only one prior ARPI treatment was allowed). At a median follow‐up of 24 months, the combination of cabozantinib and atezolizumab was superior to ARPI in terms of PFS (HR 0.65), while a non‐significant trend towards improved OS was reported (HR 0.89). However, a significant OS advantage was reported in cases with liver or bone metastasis [31].

## Solid tumor related immunosuppressive factors and their implication in PCa

In recent years there has been emerging evidence regarding the role of different immunosuppressive factors that can limit the activity of immunotherapy drugs, and this applies to most solid tumors, not only PCa. One of these is the still limited knowledge about other immune checkpoints: not only regarding their specific mechanism of action, but also regarding any interactions with the best‐known receptors, such as CTLA4 and PD‐1. For example, it is known that some receptors, such as B7‐H3, have a role in the prognosis of various tumors, including PCa [35]. We highlight the still limited attention given to the role of the other ligand of the PD‐1 receptor, namely PD‐L2, which in recent years has been shown to have a predictive role in response to immune‐mediated treatment in various tumor types [36].

However, in our opinion, immunosuppressive factors common to various solid tumors should be distinguished from tumor‐specific factors. Regarding the former, it is noteworthy that the development of CAR‐T‐cell therapies has not demonstrated significant results in PCa, in line with findings in other solid tumors, suggesting that the challenge extends beyond immune checkpoints [37]. The main solid tumor‐related immunosuppressive factors are outlined below.

### TME

It has long been known that tumor cells do not proliferate in isolation but are often surrounded by a variety of stromal cells and TME factors. Typically, the TME is composed of the extracellular matrix (ECM) and various cell types: MDSCs, neutrophils, and dendritic cells, host cells, fibroblasts, and innate myeloid cells [e.g. tumor‐associated macrophages (TAMs), lymphocytes (e.g. T cells and NK cells), CAFs, and endothelial cells] [4]. Over time, the interaction between the different components of the TME is changing. Borrowing the definition from the field of ecology, these complex relationships have been described as a real ‘ecosystem’ or, as defined by Luo (2023), a ‘multidimensional and spatiotemporal unity of ecology and evolution’ [38]. In fact, just like any ecosystem in nature, both intercellular and interspecific relationships occur between tumor cells and host factors. All ‘onco‐spheres’ (primary and systemic) are part of this ecosystem and are in turn characterized by their own specific local microenvironment, which interacts in specific ways with the immune, nervous, and endocrine systems [39]. Recently, our understanding of the role of the TME in supporting tumor survival and proliferation has improved, and various TME‐targeted therapies have been generated [40]. Increasing our knowledge of the circadian clock that underlies the process of carcinogenesis and the symbiosis between tumor cells and the TME is now a cornerstone of cancer research [41]. As has been known for some years, the TME comprises endothelial and immune cells, supporting stromal cells, which plays a pivotal role in tumor survival and progression [42]. Lifestyle factors, such as diet and microbiota status, may influence components of the TME and have been correlated with tumorigenicity [43]. Although PCa is often considered immunologically ‘cold’, a subgroup of immune‐infiltrated PCa exists, but with different T‐cell signaling patterns. It is also noteworthy that the organ specificity of the TME is even more pronounced in PCa. As previously mentioned, this immunological TME changes with space and time, since the primary tumor is often ‘colder’ than metastatic disease. In PCa bone metastases, the mixed nature of lesions (osteothickening and osteolytic) drives continuous bone remodeling, which in turn inhibits the local immune responses through the secretion of transforming growth factor (TGF)‐β, together with interleukin 6 (IL‐6) released by bone marrow stromal cells [44]. Consequently, immunosuppressive mechanisms and insufficient CD8^+^ T‐cell activation contribute to an immunosuppressive TME that may help to explain the much lower efficacy of immunotherapy compared with other solid tumors [45, 46]. For these reasons, an individualized approach to immunotherapy is essential.

### TILs

It has been proven that TILs are key mediators of the immune response: in fact, high TILs correlate with improved prognosis in several solid neoplasms [47]. Previous studies conducted on patients with colorectal cancer have highlighted the potential prognostic role of TILs: in particular, Galon *et al* demonstrated that TILs have a greater ability than TNM in predicting disease‐free survival and OS in those patients [48]. Consequently, the ‘Immunoscore’ was developed, with the aim of quantifying the immune infiltrate as a prognostic classification for solid malignancies [49]. However, PCa represents one of two examples, alongside kidney cancer, in which a more prominent cluster of differentiation (CD)8^+^ T‐cell component within the TME is associated with more rapid biochemical and instrumental (e.g., on CT or PET scan) progression, as well as poorer OS [40, 50]. This prognostic difference between PCa and most other solid tumors may partly explain the limited results from studies testing novel immunotherapy approaches in PCa, even in immune‐infiltrated cases. The reason for this correlation has not yet been determined; however, it seems that a greater CD8^+^ T‐cell component is correlated with a greater co‐deletion of *RB1* and *BRCA2*, which are in turn frequently observed in cases of PCa with a worse prognosis [51]. Moreover, TILs present abnormal signaling patterns, including higher expression of PD‐1 and TIM‐3 [43]. Molina *et al* reported that the proportions of intratumoral Treg (FoxP3^+^) and T memory (CD45RO^+^) cells strongly predict PD in patients with PCa, specifically, high Treg density and low T memory density were associated with more aggressive PCa [52]. Several studies have shown that higher TIL levels are detectable in PCa cases with specific biomolecular characteristics, such as deficits in mismatch repair mechanisms [53], inactivating mutations of cyclin dependent kinase (*CDK)‐12* [54], phosphatase and tensin homolog (*PTEN*) deletion [55], and *ERG* positivity [40]. However, PCa cases with increased T‐cell infiltration remain a minority, as the typical TME in PCa is characterized by reduced T‐cell density, especially in the CD8^+^ subset [4]. Such T‐cell scarcity favors the creation of an immune‐privileged TME [56, 57], whereby immune exclusion represents the key mediator of resistance to ICIs [58]. In fact, it has been demonstrated that hypoxic PCa areas, which are characterized by low TIL density, activate an anomalous pro‐angiogenic pathway, which determines a reduced extravasation of CD8^+^ T cells [59]. In this context, even if any lymphocytes were able to reach the tumor, they would face an immunosuppressive environment [60, 61, 62]. Finally, it should be noted that TIL expression varies significantly between the primary tumor and metastases, being markedly lower in the latter [63]. Therefore, it seems reasonable to assume that, in the future, the choice of the most appropriate immunotherapy treatment will have to take into due consideration the characteristics of the specific ‘oncosphere’ (i.e., the local tumor‐immune microenvironments where tumor cells, immune cells, and stromal cells constantly communicate) in which the metastases to be treated are located, as each can influence the oncological response in a significant and unique way.

### Tertiary lymphoid structures (TLS)

In recent years, the concept of TIL has been increasingly associated with that of tertiary lymphoid structures (TLS). TLS represent intra‐ and peri‐tumoral immune cell niches with immunomodulatory activity [64]. Their formation is substantially similar to that which characterizes secondary lymphoid organs, with the difference that it develops in non‐lymphoid areas and is therefore defined as ‘ectopic lymphogenesis’ [65]. Usually favored by an acute or chronic inflammatory context, it is regulated by the TME through cytokines and chemokines, such as CXCL13, CCL19, and CCL21 [66], which attract lymphocytes towards the tumor area, allowing interactions with other cells, including fibroblasts (in particular some fibroblastic reticular cells, FRCs [67]), endothelial cells, and stromal cells, with the consequent formation of TLS. In this context, a distinctive element of TLS are high endothelial venules (HEVs), which together with dendritic cells support the formation and development of intercellular interactions within TLS [68]. The TLS structure lacks a fibrous capsule, facilitating direct contact between immune cells and tumor tissue. It does not require the presence of antigen‐presenting cells or lymphocyte migration between the neoplasm and secondary lymphoid organs, thus accelerating the development of an antitumor immune response [69].

Different TLS localization, both within and around the tumor, as well as varying degrees of maturity, significantly influence the recruitment, activation, and crosstalk between immune cells. Intratumoral TLS has proven to be an interesting prognostic biomarker in several types of cancer. Conversely, peritumoral TLS has been linked to reduced immunotherapeutic responses and poor prognosis. Furthermore, immature TLS, composed of disordered T or B cells, are often found in an immunosuppressive TME and are unable to induce strong antitumor immunity. Meanwhile, mature TLS have been associated with better prognosis in various tumor types [70]. To date, defining the exact role of TLS in PCa remains extremely challenging. However, it appears that the key issue is not so much a question of TLS presence or absence (as TLS prevalence, particularly intratumoral TLS, is relatively high in PCa), but rather their functionality. It has been demonstrated that in PCa, similarly to other tumor types [71], the presence of significant intratumoral TLS is correlated with a better prognosis, unlike the presence of peritumoral TLS, which has not been shown to have a precise correlation. Furthermore, significant intratumoral TLS are thought to be related to active or tumor‐activated neoangiogenesis mechanisms; therefore, the hypothesis of combining antiangiogenic drugs with ICIs in cases with high TLS concentrations may represent a promising strategy for the development of an effective immunotherapy in PCa. [72].

Recent studies have reported the pivotal role of genetic mutations in TLS development and maturation. For example, *TP53* and *BRCA1/2* mutations are related to increased genomic instability, which in turn promotes immune cell recruitment and TLS formation. On the contrary, *KRAS* and *BRAF* mutations can regulate TLS density and maturity. Moreover, patients with intratumoral TLS harbor a lower *SPOP* mutation rate and a higher *TP53* mutation rate [73], suggesting that specific genetic alterations may foster an immune‐permissive TME.

Several studies have demonstrated differences between intratumoral and peritumoral TLS also in terms of immune checkpoint expression: in particular, peritumoral TLS express higher PD‐L1 and lower CD8 levels compared with intratumoral TLS, consistent with an immunosuppressive TME [74]. Moreover, peritumoral TLS are characterized by higher Tregs infiltration, which in turn suppresses dendritic cell and T‐cell function within TLS [72].

### B lymphocytes

Growing evidence supports the concept that B cells play a key role in immune modulation of the TME. They can both promote and counteract tumor development, and the prevalence of one function or another depends on several factors, including tumor type and specific local TME. Within an inflammatory microenvironment, as well as in the context of a TME, B lymphocytes are often located within the TLS, a feature that appears to correlate with improved prognosis and responses to immunotherapy across multiple tumor types [75].

Compared with benign prostatic hypertrophy, PCa demonstrates a greater infiltration of B lymphocytes within its TME, which is directly proportional to the aggressiveness of the neoplasm [58]. B lymphocytes secrete lymphotoxin, which activates *BMI1* and *IKKα–STAT3* signal transduction pathways in PCa cells, promoting the onset of CRPC and facilitating tumor progression [76,77]. Furthermore, IgA class‐switched IL10^+^PD‐L1^+^ plasma cells can limit the efficiency of immunotherapy by suppressing CD8^+^ T‐cell activation in a TGFB‐dependent manner [61]. The role of plasma cells versus tumor cells is sometimes controversial, likely depending on the type of cytokine secretion within the specific TME. Wiener *et al* reported that higher concentration of plasma cells, NK cells, and increased IgG expression were associated with a more inflammatory phenotype and prolonged recurrence‐free survival, particularly among patients of African‐American ethnicity [78].

However, TIL‐B cells may also correlate with poor prognosis due to the presence of regulatory B cells (Bregs), which promote immune tolerance [79]. Unlike Tregs, which express *FOXP3*, Bregs are a heterogeneous cell set, among which several IL‐10 and IL‐35 expressing subsets have been discovered. For example, plasmablasts (CD138^+^CD44^Hi^), plasma cells (CD138^+^MHC‐II^Lo^B220^+^), and Tim‐1^+^CD19^+^ B cells were demonstrated to release IL‐10 and modulate immune suppression in mice [75]. Moreover, Shen *et al* identified a subset of IL‐35 producing B cells, which were associated with shorter survival after primary and secondary infection in mice, suggesting the role of IL‐35^+^ Bregs in facilitating anti‐inflammatory pathways, and consequently in preventing the onset of immune‐related adverse events during and after immune therapies [80]. A subset of TGF‐β1‐expressing Breg cells has been shown to suppress immune responses in gastric cancer by converting CD8^+^ cells into CD4^+^Foxp3^+^ Treg cells in a TGF‐β‐dependent manner [81]. TGF‐β secretion by this subset of Breg cells is thought to be due to several factors, including growth factors (e.g. phosphatidylinositol glycan biosynthesis class F protein—PIGF) or the pathway involving the BCR, TLR, and CD40 receptors [82]. The presence of CD1d‐expressing Bregs has been reported, capable of activating a positive feedback loop with natural killer T cells, which in turn stimulate proliferation and maturation of B cells by secreting some cytokines, such as IL‐4 and IFN‐γ [82]. Furthermore, some Breg subsets expressing immune checkpoint inhibitors such as PD‐L1 and Tim‐1 have been described. PD‐L1^+^Bregs have been shown to curb proliferation of CD8^+^ T cells, CD4^+^CD25^−^ T cells, and CD49b^+^NK cells in a murine model by limiting IFN‐γ and tumor necrosis factor (TNF)‐α secretion, thereby promoting tumor growth in breast cancer models in mice [83]. PD‐1 and PD‐L1 expression has been demonstrated to promote immunosuppressive functions in specific B‐cell subgroups, in several solid tumors [84] (the same is also true for Tim‐1^+^ Bregs [85]), and their presence has been found to be directly proportional to the neoplastic stage and disease progression. It is believed that this heterogeneity of Bregs arises from different stages of B‐ cell development, driven by specific factors, including the specific oncosphere; consequently, different tumor contexts may drive activation of one or more subgroups of Bregs [86]. Some studies also suggest that even the location of these cells (i.e., peripheral blood versus intratumoral) may contribute to variation in the Breg phenotype [87]. In this regard, several studies have suggested the hypothesis that any B cell can, under certain conditions, become a Breg [88].

It is known that Bregs can inhibit T cells through the production of adenosine [89]. Moreover, Bregs are associated with increased Treg frequency and suppress cytokine secretion by CD4^+^ T cells (including IFNγ and TNFα); however, understanding of the entire crosstalk mechanism between these two lymphocyte populations remains incomplete [80]. Since increased Breg frequency in TME is correlated with inhibited immune responses against cancer cells, various attempts are underway to hamper/govern the action of Bregs to reactivate the antitumor immune response [90].

### MDSCs

The acronym MDSC was coined in 2007 to indicate a group of immature cells with immunosuppressive potential, generated under emergency situations, induced by an altered leukopoiesis such as during inflammatory processes or in tumors. These cells can accumulate in conditions of prolonged or chronic stress. Although molecular markers characterizing MDSCs are now known, several subsets have been discovered based on differential expression of one or more of these markers: usually the HLA‐DR^−^ Lin^low/−^ CD33^+^ CD11b^+^ phenotype is common to most subsets; among them, Lin^−^ HLA‐DR^−^ CD33^+^ cells constitute one of the most immature MDSC populations [91].

Multiple mechanisms promote cytokine upregulation, thereby contributing to MDSC recruitment in the TME, including driver‐gene mutations (e.g. loss of *SMAD4* [92]), hypoxia (e.g. via CX3CR1 [93], VEGFR3 [94], or Arg‐1 [95] signaling), PGE2 [96], and interleukin (namely, IL‐17 and IL‐1β) overexpression [97]. Moreover, other molecules, such as stromal cell‐derived factor‐1 (SDF‐1, alias CXCL12) [98], NF erythroid 2‐related factor 2 (Nrf2) [99], and high mobility group box protein‐1 (HMGB1) [100] contribute to MDSC accumulation in the TME, by inhibiting apoptosis. Inhibition of apoptosis implies that the TME impairs MDSC maturation, enabling MDSCs to act as sustained inhibitors of the immune response against the tumor: consequently, their presence in the TME is correlated with poorer prognosis [91].

Preclinical studies have shown that MDSCs can promote tumorigenesis across multiple histologies through several mechanisms: (1) promotion and expansion of Tregs by modulating indoleamine‐2,3‐dioxygenase (IDO) [101] or by upregulating PD‐L1 and CD86 [102]; (2) enhancement of Breg activity [103]; (3) inhibition of CD8^+^ T cells through multiple mechanisms including: (a) production of molecules such as nitric oxide and reactive oxygen species (ROS); (b) a possible perforin‐ and granzyme‐dependent manner; and (c) amino acid depletion [91]. However, the effects of MDSCs are not limited to the promotion of a pro‐tumorigenic TME, but also influence other factors, such as modulation of the microbiota, and promotion of chronic non‐tumoral inflammatory conditions, which may further contribute to the proliferation of the premetastatic niche [91]. Indeed, MDSCs may contribute to tumor progression by influencing: (1) stemness, for example (a) secretion of exosomes expressing high concentrations of S100A9 [104]; (b) by production of IL‐6, which through interacting with STAT3 confers stem cell‐like properties to tumor cells [105]; (2) angiogenesis, through production of adenosine mediated by CD73 expressed on the MDSC membrane [106]; and (3) invasiveness, in an NF‐kB2‐dependent manner [107].

In PCa, MDSCs are also involved in endocrine resistance. In fact, MDSCs secrete IL‐23, which activates the AR pathway, increasing survival and disease progression in androgen‐deprived conditions. Blood and neoplastic samples from patients with CRPC show increased IL‐23 levels alongside an enrichment of MDSCs within the TME. Consequently, inhibition of IL‐23 can reverse resistance to androgen deprivation therapy (ADT) in PCa [108]. Thus, targeting either MDSCs or IL‐23 may be an effective strategy against PCa. In this regard, it is worth noting that the CD33 targeted antibody–drug conjugate gemtuzumab ozogamicin has been approved for use in patients with CD33^+^ acute myeloid leukemia; however, its efficacy against PCa MDSCs remains to be demonstrated [109].

### Macrophages

Another important cellular component within PCa‐TME are macrophages. These cells are known for their ability to activate or inhibit the immune response based on their double phenotype, classic (M1‐pro‐inflammatory) or alternative (M2‐immunosuppressive). Different factors may contribute to the prevalence of one or other component. In particular, IFN‐γ or LPS promotes the pro‐inflammatory M1 phenotype, whereas IL‐4, IL‐6, IL‐13, SDF‐1, and efferocytosis activate the immunosuppressive M2 phenotype [110, 111]. As evidence of this, over‐representation of M2 macrophages in the PCa TME has been correlated with extracapsular extension and rapid biochemical progression [112]. M2 macrophages are also thought to be involved in the development and maintenance of a protumor oncosphere in bone, thereby contributing to the progression of PCa metastases at that site [46].

Metastasis‐associated macrophages are a specific subgroup of macrophages derived from circulating inflammatory monocytes and characterized by specific cell membrane markers, such as CD204 and IL‐4 receptor (IL‐4R). The latter contributes to metastatic growth, whereas its absence results in inhibition of metastatic growth [113]. Depletion using clodronate liposomes and colony‐stimulating factor 1 receptor (CSF1R) reduces M2‐macrophage populations and inhibits cell growth in breast cancer bone metastases [46]. However, certain subsets of tumor‐associated macrophages, namely SPP1^hi^‐TAMs, increase in number as PCa progresses, suppress the activity of CD8^+^ T cells, and do not respond to anti‐CSF1R antibodies. The activity of these cells is thought to be regulated by the adenosine pathway; indeed, pharmacological inhibition of the adenosine A2A receptor dramatically reduces Spp1^hi^‐TAM‐mediated immunosuppressive action against CD8^+^ T cell activity *in vitro* and enhances the efficacy of PD‐1 inhibitors in patients with CRPC *in vivo* [114].

### Neutrophils

As with other immune cell subtypes, such as T helper (Th) cells and macrophages, neutrophils can also present a different phenotype, namely anti‐ (N1) or pro‐tumorigenic (N2), based on various stimuli, particularly cytokine signals, including G‐CSF, IL‐1β, and TGF‐β [115, 116]. Murine studies have demonstrated a role for neutrophils in promoting tumor progression [4]. However, the susceptibility of neutrophils to cytokine stimuli, together with the progressive ability of PCa during its natural history, to suppress the cytotoxic capacity of neutrophils, in particular by reducing the formation of extracellular traps [117], makes the development of ‘*ad hoc*’ antitumor therapies particularly complex at present.

### CAFs

It is now known that CAFs play a pivotal role in regulating TME functions: indeed, this subgroup of stromal cell promotes not only tumor progression, but also the establishment of an immunosuppressive TME [118]. CAFs have been reported to upregulate glutathione synthesis and reduce the production of ROS, consequently leading to chemoresistance. Sun *et al* demonstrated that chemotherapy determines the secretion of WNT16B from CAFs, thus contributing to the development of chemoresistance and progression [119].

Furthermore, CAFs can reduce the efficacy of ADT by secreting pro‐neuregulin‐1 (pro‐NRG1), which in turn activates the HER‐3 pathway. In support of this, the highest concentrations of pro‐NRG1 have been detected in patients with PCa with a poorer prognosis [120]. CAFs can also counteract ADT through an additional paracrine mechanism whereby these cells promote glucosamine‐mediated upregulation of 3β‐hydroxysteroid dehydrogenase‐1 (3βHSD1), thereby contributing to the development of CRPC [121].

However, the inhibitory activity of CAFs is not only directed towards hormone therapy. A recent study has shown that a higher percentage of CRPC‐CAFs was directly correlated with reduced sensitivity to immune checkpoint inhibitors [122]. Several studies have demonstrated that CRPC‐CAFs can inhibit the activity of immunotherapy through multiple mechanisms including WNT2‐mediated impairment of dendritic cell function [123], or TGF‐β‐mediated suppression of CD8^+^ T cells [124]. Moreover, CAF proliferation contributes to the formation of a fibrous stroma, resulting in local hypoxia and chronic inflammation, which is further exacerbated by alterations in the local vascularization [125]. Therefore, CAFs contribute to stiffening of the ECM, which then becomes a physical barrier to immune cell infiltration and their access to tumor cells, with a consequent reduction in cytotoxic antitumor capabilities [126].

### Matrix metalloproteinase 2 (MMP2) and exosomes

MMP‐2 is a type IV collagenase, frequently expressed in PCa cells and associated with poorer prognosis [127]. MMP‐2^+^ PCa cells acquire the ability to cross the basement membrane and ECM and enter the bloodstream, whereas MMP‐2^−^ cells can disseminate in a stacked fashion through fissures created by MMP‐2^+^ cells. The interactions between tumor and stroma triggers phenotypic reprogramming of both cell types [127]. In this context, MMP‐2‐mediated degradation of the extracellular matrix leads to proteolysis of cytokines and their receptors, such as IL‐2R, IL‐6R, and TNF receptors [128]. MMP‐2 mediates cleavage of the IL‐2R‐alpha receptor, thereby promoting apoptosis of cytotoxic T cells. Furthermore, MMP‐2 induces Th2 polarization of Th1 subtype macrophages, resulting in modulation of the antitumor immune response [129].

However, the immunosuppressive actions of MMP‐2 are not limited to local effects: MMP‐2 can also have a systemic effect through the production of exosomes. These are membrane‐bound vesicles measuring approximately 50–100 nm in diameter, which fuse with the outer membrane of the tumor cell and are released into the bloodstream via exocytosis. Exosome membranes express a diverse range of integrins, potentially playing a role in the organotropism of metastases [130]. Moreover, exosomes are believed to be involved in the mechanisms of intercellular interaction between tumor cells and cellular recruitment into the TME [131]. In fact, by fusing their membranes directly with those of target cells, they can influence the plasma membrane content of TME cells, altering immune function in two main ways [132]: (1) by promoting, at the bone marrow level, the differentiation of monocytes into MDSCs and their subsequent migration towards the tumor, contributing to the formation of an immunosuppressive TME [133]; (2) by influencing lymphocyte activity: specifically, exosomes contribute to reducing the cytotoxic activity of T lymphocytes and NK cells, and conversely to increasing the proliferation of Tregs and Bregs [134, 135], thus exerting an immunosuppressive effect on the so‐called ‘pre‐metastatic niche’, which therefore becomes an ideal starting point for the implantation of circulating tumor cells at future metastatic sites [126].

### Circadian clock‐mediated regulation of neoplastic cells and TME crosstalk

The term ‘circadian rhythm’ refers to the phenomenon by which various living organisms regulate the cyclicity of physiological, behavioral, and biochemical functions [136]. The basis of the entire circadian mechanism is a central clock, in the anterior hypothalamic suprachiasmatic nucleus (SCN), which sends signals to peripheral clocks in the body, regulating their functions through transcription‐translation feedback loops [137, 138]. In particular, certain molecules, such as circadian locomotor output cycles kaput (CLOCK) and brain and muscle ARNT‐like protein‐1 (BMAL1), act in two ways: (1) via binding to E‐box motifs, they regulate the expression of transcription inhibitors, including period circadian regulator 1 (*PER1*) and period cryptochrome circadian regulator 1 (*CRY1*) genes. The proteins derived from the expression of these genes form a molecular complex that inhibits the CLOCK‐BMAL1 complex. (2) They also regulate the expression of nuclear receptors REV‐ERBα/β and retinoic acid receptor‐related orphan receptors (RORs), which can inhibit the action of the CLOCK‐BMAL1 complex [138]. Emerging evidence from human studies has highlighted the correlation between disruption of the circadian clock (e.g. due to shift work [139] or eating habits, such as so‐called ‘late eaters’ [140]) and increased risk of various diseases [141, 142], including metastasis in several malignancies [143]. However, as far as PCa is concerned, it must be specified that the interaction between night work and the onset of cancer is still a matter of discussion, given that some authors, while recognizing the role of the circadian rhythm in regulating the production of androgen and AR, do not highlight a significant influence on the risk of neoplastic transformation [144, 145, 146]. Circadian clocks exert influence on tumorigenesis and tumor progression by managing several cancer hallmarks [147, 148], including DNA damage response, genomic instability [149], mitosis, apoptosis, senescence [143, 150], proliferation/immortalization [137, 138], and metabolism [151]. In the case of PCa, *PER1* interacts with AR determining, especially if overexpressed, a significant inhibition of growth and stimulation of apoptosis of neoplastic cells [152]. The same occurs in the case of overexpression of *PER2*, whose expression is reduced in PCa cells compared with their normal counterparts [153]. Conversely, *PER3* inhibits the stemness of PCa stem cells through the WNT/β‐catenin pathway [154]. Androgens also appear to influence the expression of *CYR1*, a gene involved in progression of PCa as it is implicated in the circadian regulation of DNA repair mechanisms [155]. Regarding the role of circadian rhythm genes within the TME, it has been demonstrated that such genes (e.g. *CLOCK*, *ARNTL*, and *PER3*) also influence (1) inflammation, through the recruitment of macrophages, neutrophils, lymphocytes, and dendritic cells [150, 156]; (2) immune escape mechanisms, through the recruitment of lymphocytes and the expression of immune checkpoint molecules, including CTLA4 and PD‐L1 [41, 58]; (3) tumor progression and metastasis, through a depletion of *ARNTL* in macrophages, which leads to downregulation of mitochondrial metabolism and nuclear factor erythroid 2‐related factor 2 (NRF2), with consequent production of pro‐inflammatory cytokines [157, 158].

Furthermore, studies in murine models have confirmed the prominent role of circadian clocks in regulating the antitumor efficacy of radiotherapy [159] and chemotherapy [160, 161].

### Immune checkpoints

Some recent studies have suggested that the aforementioned cGAS–STING axis‐mediated innate immunity [14] and the downstream checkpoint ligands could represent potential immunotherapy targets for PCa:

### Programmed death ligand 1 (PD‐L1)

PD‐1 and its ligands, PD‐L1 and PD‐L2, play a key role in supporting self‐tolerance under physiological conditions by modulating T‐cell activation in peripheral tissues. Unlike other tumor histotypes, the reported expression frequencies for primary PCa varies greatly, ranging from 8% to 92% [7, 53, 162, 163], while data for stage IV disease remain even more limited. There are several reasons for these discrepancies: differences in sample processing, antibody selection, and their scoring system [63]. Furthermore, as previously mentioned with regard to TILs, PD‐L1 expression can vary significantly between the primary tumor and different metastatic sites: in fact, Szekely *et al* evaluated the expression of various immune checkpoint inhibitors in breast tumors and found reduced PD‐L1 expression in metastases compared with primary tumors. However, when distinguishing between metastatic sites only, PD‐L1 expression at the lymph node level was observed to be higher than that expressed by other metastatic sites, such as bone metastases [164]. This further confirms that specific local oncospheres strongly influence immune expression, and consequently responses to immunotherapy. Therefore, before proceeding with further testing of ICIs, a comprehensive evaluation of PD‐L1 expression in these tumors would be appropriate. We have previously mentioned the work of Bishop *et al* who demonstrated increased expression of PD‐L1 in patients previously treated with enzalutamide, suggesting a role of hormone therapy in inducing its expression [11]. In confirmation of this, Haffner *et al* demonstrated that the transition from the hormone‐sensitive to castration‐resistant PCa (CRPC) may induce greater expression of PD‐L1 [10]. However, another study demonstrated a trend towards a lower expression of PD‐L1 among patients treated with neoadjuvant hormone therapy [53].

### PD‐L2

For several years, most research has neglected PD‐L2, focusing mainly on the role of PD‐L1: in fact, this choice was linked to the belief that PD‐L2, unlike the widespread expression of the first ligand, was expressed only on macrophages and other subtypes of antigen‐presenting cells. However, more recently, it has been demonstrated that PD‐L2 is expressed across different tumor histotypes, and that its expression has a predictive role in treatment response, independent of PD‐1 inhibition [165]. Physiologically, the binding affinity of PD‐L2 to its receptor is much stronger than that of PD‐L1, resulting in different signaling. The expression of PD‐L1 suggests the activation of a mechanism that opposes tissue damage during inflammatory processes, unlike the expression of PD‐L2. This difference seems to date back to placental mammals, with the aim of maintaining immune tolerance in the event of inflammation [166]. Unlike PD‐L1, which is poorly expressed in PCa cells, PD‐L2 expression is higher, particularly in more aggressive PCa [167]. Its expression is known to correlate with immune‐related pathways, thus such overexpression suggests an important role for PD‐L2 in the immune response of PCa patients. Wang *et al* [168] reported a correlation between soluble PDL2 (sPDL2) and higher risk of biochemical recurrence in patients with PCa. Moreover, PD‐L2 overexpression has been significantly associated with radiation, suggesting that it may have a role in future combination strategies with radiotherapy [167]. To date, no drugs specifically targeting PD‐L2 have been approved, however anti‐PD1 immunotherapeutic agents, such as pembrolizumab, are able to inhibit the binding of PD‐1 to both its ligands, including PD‐L2, which may justify their use in combination approaches, for example with radiotherapy in tumors overexpressing this ligand.

### CTLA‐4

CTLA‐4 is a T‐cell membrane molecule that competes with the costimulatory receptor CD28 for binding to CD80/CD86 ligands expressed on antigen‐presenting cells (APCs) [169]. This interaction delivers an inhibitory signal that suppresses T‐cell proliferation and IL‐2 secretion [170]. Zhang *et al* demonstrated that, in patients with PCa, CTCs express CTLA‐4, a checkpoint that is known to be expressed on the plasma membranes of many cell types, including immune cells, pituitary cells, and some tumor cell lines. The same authors also demonstrated that CTLA4, like the two PD‐1 ligands, is expressed only in a subset of CTCs and only at certain timepoints [171].

### B7‐H3 and B7x

It has long been known that B7 family members, in particular B7‐H3 and B7x, are correlated with disease progression and poor OS in patients with PCa [172].

Zhang *et al* have suggested that B7‐H3 may represent a significant target for immunotherapy since it is almost universally expressed on CTC cells in every PCa cohort and at every stage. Its expression, even in PD‐L1‐deficient CTCs in patients with mCRPC treated with an anti‐PD‐1 therapy, suggests a potential mechanism of immune evasion mediated by B7‐H3 [171].

Other studies report the correlation between B7‐1 (CD80), a ligand for CTLA4 and CD28, and prognosis in patients with PCa. [173]. Kwon *et al* demonstrated that ectopic CD80 expression in a cell line pTC1 grafted into an *in vivo* murine model could enhance immune destruction of PCa cells [173]. However, Wang *et al* demonstrated a consistent association between cellular and circulating forms of CD80 [174]. In particular, they reported that human sCD80, produced through alternative splicing of the gene, can inhibit activation and proliferation *in vitro* of T lymphocytes [175]. Consequently, sCD80 can enhance T‐cell co‐inhibition due to its linkage with CTLA4, leading to PCa immune escape and increased risk of recurrence [172]. Moreover, sCD80 competing with its membranous isoform, may regulate its costimulatory effects on lymphocytes. However, further investigations are needed regarding the role of sCD80 in PCA, both as a biomarker and as a potential therapeutic target.

### V‐type immunoglobulin domain‐containing suppressor of T‐cell activation (VISTA)

VISTA is a type I transmembrane protein comprising a single N‐terminal immunoglobulin (Ig) V domain, linked by a stalk to a transmembrane domain terminating in a cytoplasmic tail [176]. VISTA is overexpressed on myeloid cells, particularly macrophages, conventional dendritic cells, monocytes, and circulating neutrophils, but also on CD4^+^ T cells, in particular on naïve cells and Tregs and, to a lesser extent, on memory CD4^+^ T cells, CD8^+^ T cells, and NK cells, while it is not expressed on B cells [177]. VISTA also represents a downstream target of p53 activity in response to cellular stress, suggesting that its expression is also activated in apoptotic cells following DNA damage [178].

Gao *et al* demonstrated that in patients with prostate cancer, treatment with ipilimumab significantly increased VISTA expression across several immune cell subsets, including CD4^+^ T cells, CD8^+^ T cells, and CD68^+^ macrophages. Specifically, VISTA was expressed on CD4^+^ (4%) and CD8^+^ (7%) T cells only post‐treatment, whereas it was also expressed on CD68+ macrophages before therapy, but at percentages four‐fold lower than post‐treatment (7% versus 31%); VISTA expression was usually independent of PD‐L1 expression, so much so that only 2% of macrophages after ipilimumab therapy coexpressed both immune checkpoints. Both types of macrophages, PD‐L1^+^ and VISTA^+^, also coexpressed a higher post‐treatment percentage of CD163 and Arg1, indicative of an M2‐like phenotype and function, respectively. VISTA expression was also observed to be increased on both tumor cells and circulating monocytes. VISTA expression correlated with reduced cytokine secretion, in particular, IFN‐γ and TNF‐α [179]. The study findings challenge the hypothesis that PCas are necessarily ‘cold’ tumors that do not respond to immune checkpoint inhibitors: on the contrary, they may become immune sensitive, but the CTLA4 and PD‐1/PD‐L1 pathways may not be the only relevant or sufficient checkpoint axes [180].

### STAT3

The STAT3 signaling pathway has a role in regulating function of immunosuppressive cells and inhibition of dendritic cells into the TME. Consequently, the STAT3 pathway can promote immune escape mechanisms in tumors [181]. Furthermore, STAT3 modulates secretion of immunosuppressive molecules by the neoplastic cells themselves [182]. For these reasons, STAT3 has been suggested as a promising target in various neoplasms [183]. In this regard, galiellalactone, a STAT3 inhibitor, has been shown to be able to block MDSC development and inhibits the production of cytokines such as and IL‐1ß, IL‐6, and IL‐10 from monocytes, as well as GM‐CSF and IL‐8 production from PCa cells [184]. GPB730, a semi‐synthetic analogue of galiellalactone, has been shown to limit the immunosuppressive action of NK cells and lessen PCa cell‐dependent pSTAT3‐S727 expression, both in human PCa cells and in immune cells [185]. Therefore, the immunosuppressive action of STAT3 inhibitors on the tumor microenvironment suggests that it may also favor the effect of ICI treatment. In fact, as has been reported in murine models [186] and in human studies [187], inhibition of STAT3 enhances the anti‐tumor efficacy of anti CTLA‐4 treatment in PCa.

## Microbiota

In recent years, there has been an increasing awareness in the scientific literature of the role of the intestinal microbiome in tumorigenesis processes, including PCa, and its treatment. The microbiome–PCa interaction is usually divided into two subgroups: direct and indirect. The first subgroup includes processes that directly involve the microbiome and the organ of origin of the primary tumor; the second includes processes that involve the entire gastrointestinal tract [188].

Recently, Huang *et al* reported a reduction in alpha‐diversity of the gut microbiota in PCa cases compared with the control group. These differences concerned phyla (with a higher percentage of Proteobacteria compared with Actinobacteria, Bacteroidetes, and Firmicutes which were therefore relatively lower in PCa cases) and genera (with a higher presence of *Prevotella*, *Escherichia*‐*Shigella*, *Faecalibacterium*, and *Bacteroides*, at the expense of *Veillonella* and *Megasphaera*) [124].

Although evidence is limited, several elements support the hypothesis that the intestinal microbiota can affect the action of oncological treatments, including radiotherapy, chemotherapy, hormone therapy (overproduction of estrogen from steroids), and immunotherapies.

With regard to the modulation of the immune response, it is known that: (1) overproduction, by the gut microbiota, of estrogens derived from steroids can enhance chronic inflammation. In particular, it has been shown that *Bacteroides massiliensis* (the most frequent bacterium in PCa cases) expresses β‐glucuronidase, which is able to deconjugate estrogens which can then bind ERs, thus promoting PCa progression [189]; (2) a diet rich in fatty acids can modify the gut microbiota, increasing the permeability of the mucosa and thus favoring the translocation of Gram‐negative bacteria into the bloodstream and mesenteric adipose tissue through the intestinal mucosa, leading to inflammation. This can lead to overproduction of IL‐6 and other cytokines at the level of the prostate tissue, favoring tumor progression [190]; (3) gut microbiota dysbiosis can promote PCa progression through TNF‐α‐mediated activation of the TLR4/MyD88/NF‐κB signaling pathway, potentially involved in α‐linolenic acid metabolism [191]; (4) the efficacy of CTLA‐4 inhibitors is modulated by the composition of the microbiota (*Bacteroides fragilis* and/or *Bacteroides thetaiotaomicron*, and *Burkholderiales*), through effects on IL‐12‐dependent Th1 immune responses [192]; (5) regarding the PD‐1/PD‐L1 axis, it has been demonstrated that oral administration of *Bifidobacterium* as a monotherapy during treatment with anti‐PD‐L1 improved antitumor responses, almost completely blocking tumor growth in murine models. In support of this result, a concomitant increase in dendritic cell functionality was also found, with a consequent accumulation of CD8^+^ T cells in the TME [193].

To date, however, studies on the microbiome composition have led to conflicting results and therefore their role in PCa tumor regenesis is still unclear, partly also due to methodological differences in conducting research.

Consequently, the therapeutic implications of these potential interactions remain poorly understood, and still little studied. It is hoped that the increasing use of advanced sampling techniques together with computational methods may facilitate research in the near future.

## PCa‐specific immunosuppressive factors

In addition to considering the role of the TME, there are several factors specific to PCa that may have contributed to the poor results obtained using the new immunotherapy treatments. The main factors are listed below.

### Biomolecular subtypes of PCa

Although this is already a reality for other tumor types, the lack of an accurate biomolecular classification for PCa currently represents a further limitation that undermines the overall evaluation of the activity of the immune response for this histotype. However, some genomic alterations reported in the literature may render some PCa subtypes more aggressive and consequently more immunosensitive.

### *PTEN* deficiency

Among all gene mutations, *PTEN* deficiency (usually biallelic loss, sometimes also point mutations, gene rearrangements, and epigenetic changes) represents the most frequent in PCa, ranging from an incidence of 20% in non‐metastatic cases up to 40% in mCRPC cases [194]. In addition to being a known antagonist of the phosphoinositide 3‐kinase (PI3K)–Rac‐alpha serine/threonine‐protein kinase (AKT)–mammalian target of rapamycin (mTOR) pathway function, *PTEN* has been shown in recent years to also have an important role in modulating the immune response [55]. Several preclinical studies have linked *PTEN* deficiency to a chronic inflammatory and immunosuppressive state due to MDSC recruitment [111, 167]. This can occur through various strategies, including (1) activation of the NF‐κB pathway, particularly in *Pten* ^PC−/−^; *Zbtb7a* ^PC−/−^ tumors [62] and (2) recruitment of immunosuppressive Treg cells and M2 macrophages in *Pten* ^PC−/−^; *Trp53* ^PC−/−^ tumors. This correlation also seems to be associated with increased expression of indoleamine 2,3‐dioxygenase 1 [195]. Regarding Tregs, a significant increase was observed in *PTEN* ^−^ prostate cancer, although this depended on the sampling site. Specifically, an increase in Tregs was observed in liver metastases and in bone metastases, the latter being associated with a decrease in cytotoxic T cells, while in lymph nodes, an increase in cytotoxic T cells was observed [129]. These discordant alterations in immune function are further evidence of the crucial role of the TME, with *PTEN* loss resulting in divergent changes based on the surrounding tissue context.

Further confirmation of the immunomodulatory activity of the *PTEN* gene has been evidenced by (3) the ‘immune desert’, condition observed in *Pten* ^PC−/−^; *Pml* ^PC−/−^ tumors [62].

Other studies have shown that *PTEN* is implicated in cellular senescence mechanisms, which in healthy cells represent a further mechanism for the elimination of any neoplastic cells [196]. In particular, it has been demonstrated that *Pten* deletion leads to increased survival of murine cells, particularly in conjunction with the deletion of *Chd1* or *Erk5*, likely due to lower chemoattraction of MDSCs and a consequent greater infiltration of T cells into the TME [167]. Furthermore, *PTEN* deletion promotes the expression of CXCL molecules [197], which in turn accentuate the CXCR1/2‐mediated chemotaxis of macrophages towards the TME, resulting in inhibition of cellular senescence and prolonged tumor survival [198]. As reported, the development of drugs blocking the CXCL–CXCR pathway may represent an interesting research opportunity particularly in the case of *PTEN* deficiency. However, it must be emphasized that such strategies should also consider inhibition of the *STAT3* axis pathway. It has been demonstrated that activation of the *JAK2*–*STAT3* axis in *PTEN*‐deleted PCa can promote recruitment of Gr‐1^+^ MDSCs [199], which contribute to the establishment of a pro‐tumorigenic TME phenotype [110].

Finally, several studies have demonstrated an interaction between *PTEN* deletion and changes in the tumor stroma, with increased proliferative signaling that favors tumor cell growth. This may occur through different mechanisms: (1) overexpression of CXCL8, capable of causing both autocrine hypersecretion of CXCR4/CXCR7 and CCR2 by tumor cells and paracrine secretion of CXCL12 and CCL2 by stromal cells [197]; (2) dysregulation of AKT, with consequent autocrine overexpression of CXCL12/CXCR4 by tumor cells [200]; (3) overexpression of early growth response‐1 (EGR‐1), a regulator of angiogenesis and osteoclast activity in bone metastases [55].

### dMMR

dMMR is present in approximately 3–12% of PCas [201]; due to its association with tumor hypermutability and neoantigen generation, this condition represents a promising candidate for immunotherapeutic approaches. This has also been studied in other tumor contexts, where dMMR is correlated with a favorable prognosis [202]. Unlike these tumors (such as colon cancer), in PCa the dMMR status correlates with a higher Gleason grade and a worse prognosis, underscoring the peculiarity of this histotype [203]. In studies that have tested immunotherapeutic agents in PCa with dMMR, objective responses have been reported in approximately 50% of cases. However, in other patients, ICI‐based treatments have shown a detrimental effect, suggesting the involvement of other factors that are not fully understood [203]. One possible explanation is that a significant percentage of dMMR PCa lacks CD4^+^ and CD8^+^ T‐cell infiltration or PD‐L1^+^ cells – unlike other histotypes and the majority of PCa presenting with the same deficit—but rather shows infiltration of M2‐polarised macrophages [204].

### Homologous recombination‐deficiency (HRD)

Deficits in homologous recombination (HR) mechanisms have been detected in an increasing percentage of PCas from early (less than 10% of cases) to metastatic stages, where an incidence of around 25% is reached [205]. PCa cases with HRD status (in particular, those carrying a *BRCA2* mutation) are commonly more aggressive, and less responsive to both radiotherapy and chemotherapy [206]. In line with what has been demonstrated by studies on ovarian and pancreatic tumors presenting the same genomic alteration, HRD^+^ PCas have also demonstrated sensitivity to PARP inhibitors [205]. HRD PCas present a TME characterized by the presence of several protumorigenic cells, such as lymphocytes (including CD4^+^ FOXP3^+^ Tregs, and CD8^+^ T cells) and CD163^+^ macrophages [207]. In such conditions, the use of a PARP inhibitor can activate the STING signaling pathway and increase immune infiltration with concomitant overexpression of PD‐L1. Together, these effects contribute to upregulation of the interferon signaling pathway [15].

### DNA polymerase epsilon ( *POLE* ) and D1 ( *POLD1* ) mutation

*POLE* and *POLD1* are members of the DNA polymerase family of enzymes that play a key role in DNA core strand synthesis as well as nucleotide and base excision repair. Some *POLE*/*POLD1* mutations also lead to ultramutated immunophenotypes in PCa [208], with a reported prevalence of around 0.1–0.3% of cases [209]. PCa harboring *POLE* mutations exhibit (1) very high single nucleotide variants [210]; (2) increased CD8^+^ T‐cell infiltration; (3) elevated expression of effector cytokines, similar to tumors harboring dMMR [211]. In recent years, there has been a growing literature on the predictive role of these mutations (specifically in the exonuclease ‘proofreading’ domains) in the case of ICI treatments due to their greater mutational load without microsatellite instability [174]. In particular, studies have suggested a high antitumor activity of ICIs in the presence of tumors harboring *POLE* mutations, including PCa [43]. To date, an ongoing clinical trial is testing tumors harboring *POLE* and *POLD1* mutations, without microsatellite instability, with a new anti‐PD1 antibody, toripalimab (NCT03810339; https://clinicaltrials.gov/study/NCT03810339).

### *CDK12* mutation

Mutations in *CDK12* have been found in 4.7% of PCas. Although historically linked to HR defects, the presence of this mutation determines a distinct and peculiar PCa subtype, characterized by: (1) poor prognosis, (2) high TMB, and (3) increased neoantigens (second only to PCas harboring dMMR), as well as (4) a high percentage of immunosuppressive lymphocytes (in particular CD4^+^ FOXP3^−^ T cells) within the TME [212]. In support of this, Antonarakis *et al* tested an anti‐PD1 immunotherapy on a small cohort of nine patients affected by PCa carrying a *CDK12* mutation: the authors reported a response rate of 33%, with over half of responders continuing treatment beyond 6 months [24]. More recently, in the prospective phase II IMPACT trial, Nguyen *et al* tested the combination of ipilimumab + nivolumab (cohort A) or nivolumab monotherapy (cohort C) in patients with mCRPC harboring deleterious *CDK12* alterations. The primary endpoint of a 50% reduction in PSA (PSA50) was reached in 9% of cases in cohort A and in 0% of cases in cohort C. Median PFS and median OS was 7.0 and 9.0 months in the combination cohort (A) and 4.5 and 13.8 months in the monotherapy cohort (C), respectively. These data underscore the fundamental role of TME in limiting significant responses to immunotherapy in PCa [213].

### Speckle type BTB/POZ protein ( *SPOP* ) loss

*SPOP* mutations are commonly missense mutations and have been found in 10–15% of patients with PCa [214]. In such cases, they are almost always associated with *CHD1* deletions, resulting in a distinct tumor subtype, characterized by: (1) inhibition of proteasome‐mediated degradation with increased PD‐L1 expression [171]; (2) increased presence of immunosuppressive lymphocytes within the TME; and (3) marked sensitivity to hormone therapy, even in the case of metastatic disease [215]. The dual peculiarity of this subtype, namely its marked hormone sensitivity and its high PD‐L1 expression, suggests a potential therapeutic strategy with a combination of hormone therapy and ICI. In support of this hypothesis, a combination of ADT and anti‐PD‐L1 therapy has demonstrated efficacy in mouse models [4] and, subsequently, Hawley *et al* reported encouraging data with the combination of ADT and anti‐PD1 therapy [216].

### Steroids

Another aspect to be explored is the role of steroids [2]: all patients affected by PCa who undergo treatment with docetaxel, cabazitaxel, and abiraterone acetate also undergo daily treatment (often for years) with 10 mg/day of prednisone, which is considered in international guidelines to be the maximum steroid dose recommended in patients receiving immunological therapies. However, the long‐term ‘maintenance’ usage of this dose may not be completely harmless with respect to an immunological treatment. Supporting this concern, Arbour *et al* indicated that even 10 mg of prednisone was associated with a poor outcome in patients with NSCLC [217]. Therefore, the combination of prednisone concomitantly with an immunotherapy drug may represent a sort of ‘self‐sabotage’ of the efficacy of the immunotherapy treatment.

Although in some trials, such as Keynote‐921, the dose of prednisone was 5 mg/day, it should also be considered that, in clinical practice, patients with PCa often undergo steroid treatment at a relatively higher dosage to manage symptoms associated with, for example, bone metastases [3].

### Hormonal therapy: ADT and ARPIs

Finally, an additional and often underestimated factor in modulating the immune response is the role of hormonal therapy, which is fundamental to the therapeutic algorithm for patients with PCa. Guan *et al* demonstrated that intrinsic AR activity of T cells represses IFN‐γ expression, thereby contributing a mechanism of resistance to immunotherapy [218]. Several studies have also focused on the role of ADT duration in modulating the immune response [219]: it has been shown that ADT initially enhances anti‐tumor Th1‐type responses; however, these effects are likely short‐lived and an immunosuppressive TIL landscape eventually predominates [4]. In particular, increases in CD4^+^CD25^+^FoxP3^+^ Tregs and M‐CSF1 have been reported during long‐term ADT [11]. Long *et al* demonstrated that neoadjuvant ADT determines not only a TME rich in CD8^+^ and Th1 T cells, but also overexpression of CTLA4 and PD‐L1 [219]. Erlandsson *et al* reported that neoadjuvant ADT in combination with RT determines higher levels of lymphocytes [especially, T‐bet(T‐box transcription factor TBX21)^+^ Th1 cells, CD8^+^ T cells, and CD20^+^ B cells] and CD68^+^ TAM compared with RT or ADT alone [220]. Wang *et al* demonstrated that ADT enhances TGF‐β signaling pathway‐mediated phenotypic transition of inflammatory CAFs into CRPC‐associated CAFs [221]; this condition leads to an increased paracrine secretion of SPP1, which in turn promotes ADT resistance. Similar to radiotherapy and chemotherapy, ADT modulates immune activity in a non‐specific manner [222], resulting in a concomitant increase in both pro‐ and anti‐tumorigenic mechanisms, the relative predominance of greater or lesser activity depending on the specific PCa subtype [4]. The role of ADT in promoting IL‐8 expression on prostatic epithelial cells has recently been reported. In turn, IL‐8 promotes subsequent intratumoral infiltration of pro‐tumorigenic polymorphonuclear MDSCs (PMN‐MDSCs). Importantly, the combination of an anti‐IL‐8 drug with an immune checkpoint inhibitor delayed the transition from HSPC to CRPC and increased the proportion of polyfunctional CD8^+^ T cells within the TME [223].

Other studies have focused on the influence of second‐generation ARPI therapy in suppressing adaptive immune responses [224]. Furthermore, in a previous work, we demonstrated an association between autoimmune diseases and worse prognosis in patients treated with ARPIs [225]. He *et al* analyzed single‐cell RNA sequencing data (scRNA‐seq) from pre‐ and post‐enzalutamide biopsies and observed clonal expansion of T cells [56]. These findings suggest that the response to enzalutamide (including tumor cell necrosis) may represent a ‘*conditio sine qua non*’ for eliciting a therapeutic response to a subsequent combination of enzalutamide and ICIs [226].

### Beyond ICIs: emerging efficacy landscape against PCa

The poor results obtained with the use of immunotherapies based on ICIs has prompted the search for therapeutic strategies that allow the immune system to be activated differently; in other words, to transform a TME historically considered ‘cold’ into an immunologically ‘hot’ environment. Modern research is progressing along several directions, both by improving the efficacy of immunotherapeutic drugs and by modifying the pro‐inflammatory TME, for example through the use of specific drugs involved in tumor metabolism, such as lactate inhibitors (the accumulation of lactates in fact inhibits TLS immune responses) [227] or by targeting metabolic enzymes such as indoleamine 2,3‐dioxygenase 1 (IDO1), ATP‐citrate lyase (ACLY), or carnitine palmitoyltransferase (CPT1A) [228]. One mechanism that has shown significant progress in recent years involves the development of drugs that activate immune responses through bypassing MHC expression, which is commonly suppressed in PCa as a mechanism of immune evasion (moreover, T cells are often scarcely represented into the PCa TME). In this regard, T‐cell engagers represent an ideal candidate: typically designed as bispecific antibodies; these engineered molecules simultaneously bind both CD3 expressed on T cells and tumor‐associated antigens on tumor cells. In this way, they cause direct, strong T‐cell‐mediated cytotoxicity in an MHC‐independent manner. As a result of activation, T cells secrete cytotoxic factors (e.g., granzymes and perforin) and pro‐inflammatory cytokines (like IL‐6 and TNFα) that contribute to the tumor cell lysis [229]. In this respect, they differ both from ICIs, which act on pre‐existing T cells (which, as previously mentioned, are rare in PCa TME), but also from so‐called CAR‐T cells. The latter represent an additional non‐MHC‐restricted therapeutic option, which has also been tested in PCa. The CAR molecule consists of an engineered T cell expressing on its cell membrane a tumor antigen‐binding domain fused to an intracellular signaling domain and costimulatory molecules. [37]. Aside from their different molecular structure, another fundamental difference between T‐cell engagers and CAR‐T‐cell therapy is that the latter requires longer processing times as it involves the extraction, genetic engineering, and reinfusion of the patient's T cells. In contrast to CAR‐T cells, T‐cell engagers are ‘ready‐to‐use’ molecules, which do not require customized cell manufacturing and can therefore be administered more quickly. For both CAR and T‐cell engagers, the first molecules were simpler, but showed reduced activity for various reasons, including lack of costimulatory molecules (first‐generation CAR) or short half‐life requiring continuous infusion (first‐generation T‐cell engagers). The greater complexity of later‐generation molecules (including costimulatory molecules and IL‐12 secretion to counteract the inhibitory action of the TME for new‐generation CARs [37]; Fc fragments and albumin‐binding domains aimed at prolonging the drug's half‐life for T‐cell engagers) has significantly improved therapeutic efficacy [230]. The molecules tested in studies conducted so far have targeted specific TAAs, such as PSMA, six transmembrane epithelial antigen of the prostate 1 (STEAP1) [231], delta‐like ligand 3 (DLL3—an atypical Notch pathway ligand expressed on the cell membrane in neuroendocrine tumors [232]), and kallikrein‐2 [233]. Overall, the available data to date highlight the ability of T‐cell engagers to induce biochemical and (partially) instrumental responses in patients affected by mCRPC, with ORRs ranging from approximately 10% in a study investigating the PSMA‐targeting agent acapatamab [234] to 40% in a study evaluating high‐dose STEAP1‐targeting agent xaluritamig [235].

Studies on the use of these molecules in PCa have highlighted benefits but also potential risks, including: (1) possible production of so‐called ‘anti‐drug antibodies’, with consequent potential accentuation of drug‐related toxicities and reduction of treatment activity [236]; (2) onset of cytokine release syndrome; and/or (3) neurotoxicity (immune effector cell‐associated neurotoxicity syndrome) [236]; (4) limited response durability; and (5) onset of drug resistance due to antigen escape mechanisms [230], which limit the use of such approaches only to selected patients.

## Conclusions

According to various studies, the use of immune checkpoint inhibitors against PCa has not, to date, achieved the expected results. However, the rationale for using these agents remains conceptually compelling, and a deeper understanding of pathways that regulate the immune response will allow scientific research to effectively modulate these processes.

It is also important to note the strong dependence of most studies on company‐driven choices. Consequently, the aim of demonstrating the efficacy of a given drug, often relative to the ineffectiveness of a competing but mechanistically similar drug, often prevails over the search for larger‐scale in‐depth studies on the interrelationships between the various immune checkpoints. Even in immunotherapy, a ‘TKI‐like’ approach persists, focusing on a single specific biological target; however, as we have discussed in this review, new discoveries regarding the interrelationships between tumor cells, TME, and immunotherapies should increasingly guide research towards a holistic approach, centered on the entire ‘ecosystem’ of the specific tumor.

Furthermore, the oncosphere should be viewed not as a static concept, but as a dynamic system that evolves both spatially – across primary tumors and metastases – and temporally, reflecting the clonal instability of neoplastic cells and continuous shifts in the immune balance. Continued evaluation of immunotherapeutic agents without taking these aspects into account risks yielding further disappointing results. In light of all these considerations, it will be desirable, in the coming years, to investigate through prospective studies the role of all potential factors underlying the lack of immunological response in PCa.

## Author contributions statement

GS and CL were responsible for conceptualization. GS and IT performed data curation, formal analysis, and investigation. CL supervised the study. EFG, SB, NB, ET, SZ and MCC were responsible for validation. GS, IT and CL performed visualization. GS wrote the original draft of the manuscript. GS and IT reviewed and edited the manuscript. All authors read and approved the final version of the manuscript.

## Data Availability

The data that support the findings of this study are available from the corresponding author upon reasonable request.
